# Case reports of large and symptomatic abdominal tumors in newborns: Pitfalls, difficulties in management, and surgical decision making

**DOI:** 10.1002/cnr2.1726

**Published:** 2022-10-04

**Authors:** Francesco Laconi, Charline Bischoff, Daphne Michelet, Gauthier Loron, Nathalie Bednarek, Marie‐Laurence Poli‐Merol, Maguelonne Pons, Nadia Boudaoud

**Affiliations:** ^1^ Pediatric Surgery Department Centre Hospitalier Universitaire de Reims Reims France; ^2^ Paediatric Anestesiology Department Centre Hospitalier Universitaire de Reims Reims France; ^3^ Department of Neonatology Reims University Hospital Alix de Champagne Reims France; ^4^ CReSTIC EA 3804 Université de Reims Champagne Ardenne Reims France; ^5^ Pediatric Surgery Department Centre Hospitalier Universitaire de Clermont‐Ferrand Clermont‐Ferrand France

**Keywords:** abdominal compartment syndrome, case report, neonatal surgery, neonatal tumors

## Abstract

**Background:**

Large and rapidly growing abdominal tumors may result in fatal outcomes in newborns. In some cases, a rapidly worsening clinical condition requires surgical decision‐making despite the absence of a precise histological diagnosis. In these situations, there is neither a guide nor consensus.

**Case:**

We highlight our experience with five patients with large abdominal tumors and assess the available literature for the best possible management of a rare condition.

**Conclusion:**

In these cases, laparostomy should be considered as a life‐saving procedure. If the liver is involved and coagulopathy is present, prognosis is often compromised.

## 1 INTRODUCTION

Solid neonatal tumors are rare, representing less than 2% of childhood tumors.^1^ Among them, large and rapidly growing abdominal tumors can seriously endanger the lives of newborns. There is a lack of consensus in managing these situations due to their rarity and diagnostic difficulty. Uncertainty in the nature and extent of the lesion despite well‐conducted imaging studies further question whether a correct diagnosis was made. The optimal timing for surgery remains questionable due to the possibility of the evolution of homeostasis, ventilatory state, and concerns about oncological and long‐term prognosis. In this study, we report our clinical experience with five cases to develop a decisional algorithm for handling these tumors. When surgery was inevitable, we sought to identify the choices that would be considered acceptable from medical and ethical points of view.

## 2 METHODS

With the approval of the Institutional Review Board, we conducted a retrospective study of our internal patients' database from January 2006 to December 2018 using the following search words: “abdominal mass,” “abdominal tumor,” and “abdominal compartment syndrome.” We included only infants younger than 4 weeks that underwent surgery for abdominal masses with or without an antenatal diagnosis. For each patient, we collected data concerning sex, antenatal history, birth weight, age at surgery, type of tumor, type of surgery, type of imaging and clinical results at the last control.

Table 1 shows the characteristics of the patients.

**TABLE 1:** Characteristics of the patients

	Sex	Diagnosis	Age at surgery (Days)	Type of surgery	Exams before surgery	Outcomes
1	M	Mesenteric immature teratoma	8	Laparotomy	US, MRI, AFP	Survived
2	F	Mesenteric immature teratoma	14	Biopsies, Laparotomy	US, MRI, UC	Survived, short bowel syndrome
3	F	Hepatic Haemangioendotelioma	2	Laparotomy	US, MRI AFP, UC	Survived
4	F	Adrenal Neuroblastoma	28	Laparotomy	US, UC, MIBG scintigraphy	Survived
5	M	Hepatic Multifocal Infantile Hemangioma	18	Laparostomy	US, MRI, UC, AFP	Deceased

Abbreviations: A‐FTP, alpha‐fetoprotein; MRI: magnetic resonance imaging; UC: urinary catecholamines; US: ultrasound.

## 3 CASE SERIES

Case 1 A newborn boy delivered at 39 weeks of pregnancy (WoP) + 5 days weighing 3540 g with a normal antenatal history presented with significant abdominal distension causing respiratory distress at birth. The first postnatal abdominal ultrasound (US) did not show the origin of the tumor; however, magnetic resonance imaging (MRI) showed a solid intraperitoneal mass measuring 93 × 100 × 120 mm. Due to the increasing need for respiratory support, laparotomy was performed at 8 days of life. The intraoperative findings revealed an intramesenteric tumor, which was completely resected. Pathological analysis revealed an immature grade III teratoma. Before surgery and postoperatively, alpha‐fetoprotein levels were within normal limits. At follow‐up 3 years after surgery, there was no evidence of complications or recurrence. The follow‐up was based on clinical examination, monitoring of blood markers and hepatic ultrasonography twice a year.

**FIGURE 1:**
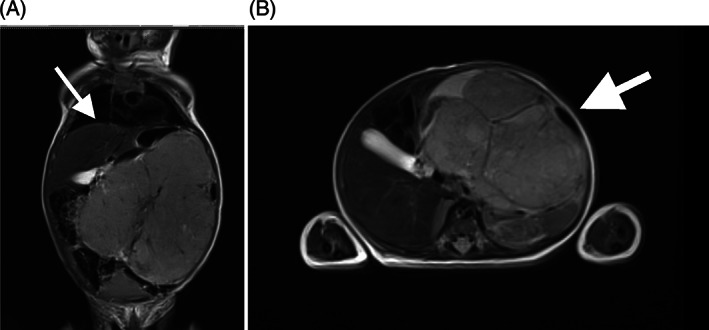
(A, B) Abdominal magnetic resonance image of an immature teratoma arising from mesenteric tissue in case 2. It should be noted that the mass compresses the intestines against the abdominal wall and significantly elevated the diaphragmatic domes, generating a compressive‐type alteration in respiratory mechanics (arrows)

Case 2 A newborn girl delivered at 37 WoP + 1 days and weighing 3600 g was revealed to have a large abdominal tumor on US the day before delivery. All previous US findings were normal. On MRI, a heterogeneous (solid and cystic) mass measuring 111 × 67 × 124 mm originating from the mesenteric root was observed (Figure 1A,B). Urinary catecholamine levels were within normal limits; however, the alpha‐fetoprotein level was remarkably elevated (358 503, 3 mcg/L, normal value <100.000 mcg/L).^2^, ^3^ US‐guided needle biopsy was performed, and pathological examination revealed an immature teratoma, grade III. Elective laparotomy was performed at 14 days of life, and the mass was completely resected. Unfortunately, on the fifth postoperative day, the peritoneal drain put in during the first surgery began to produce brown, foul‐smelling fluid, and the patient's condition suddenly worsened with fever and great abdominal distension. The naso‐gastric tube starts to give biliary fluid, and on radiography of the abdomen, clear signs of pneumoperitoneum were seen. The decision for an emergent laparotomy exploration was therefore made, revealing a massive necrosis of the small bowel that required extended intestinal resection. At the last follow‐up 17 months after surgery, she presented with well‐tolerated short bowel syndrome with no evidence of tumor relapse.

**FIGURE 2:**
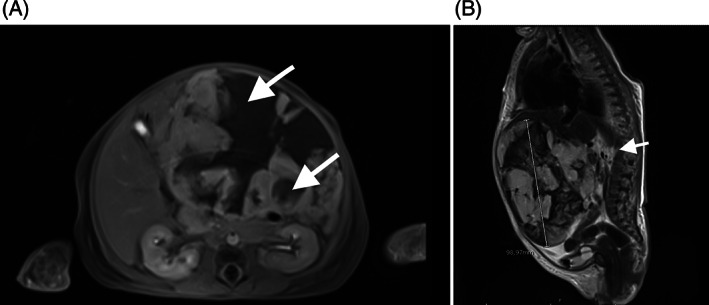
(A, B) Abdominal magnetic resonance image of hepatic hemangioendothelioma in case 3. The mass had with necrotic areas in the center (arrows). The hepatic origin of the mass is unclear in the images, which instead shows it originating from the retroperitoneum

Case 3 A newborn girl delivered at 34 WoP and weighing 3850 g with a normal antenatal history presented with a large abdominal mass at birth. Alpha‐fetoprotein and urinary catecholamine levels were within normal limits. The MRI measured a solid mass of 11.5 × 75.5 × 99 mm, revealing areas of central necrosis, which was postulated as responsible for acute progressive respiratory distress (Figure 2A,B). The delay in the onset of respiratory signs, lack of chest retraction, and the absence of alveolar syndrome on lung radiography, were not in line with a hyaline membrane disease. Oppositely, x‐ray revealed an abdominal distention and ascension of the diaphragmatic domes. Determining the exact origin of the tumor was not feasible even after an expert review of the MR images. The increasing need for respiratory support and severe coagulation disorder (requiring platelet and plasma transfusions on day 1 and 2, 15 and 20 ml/kg, respectively) prompted decompression surgery at 2 days of life. During surgery, the mass, which originated from the left liver lobe, was completely resected. Pathological examination revealed hepatic hemangioendothelioma. The doses of thyroid hormone supplementation (fT3, TSH, and fT4) after surgery were in the following ranges: fT3 = 2.15 ng/L (N: 1.95–6.04), fT4 = 17.9 ng/L (N: 8.9–22.0); TSH = 1.60 mUI/L (N: 0.72–11.00). No evidence of recurrence was observed at the last follow‐up 3 years after surgery.

Case 4 A newborn girl delivered at 40 WoP and weighing 3030 g with a normal antenatal history was admitted to the neonatal intensive care unit for cardiogenic shock, acute respiratory distress, and hypertension (123/78) 3 h after birth. Cardiomegaly was noted on the chest radiograph, and abdominal US revealed a left adrenal mass measuring 41 × 40 × 42 mm. Nicardipine was initiated immediately at 1 μg/kg/min, allowing for blood pressure correction (89/66 mmHg) and progressive resolution of the hemodynamic status. Nicardipine was successfully shifted to oral nifedipine on the sixth day of life.

Vanillylmandelic acid (VMA) and homovanilic acid (HVA) were measured in the urine on the first day of life. After 15 days, the results revealed that the VMA and HVA levels were 50.19 mmol/L (normal, <15.50) and 10.17 mmol/L (normal, <18.80).

On MIBG scintigraphy, no metastatic tumor was observed in the suprarenal region. The patient was referred to the national staff of pediatric oncology, and the decision for surgical removal was made. The patient underwent open surgery at 28 days of life. Pathological examination of the tumor revealed an adrenal neuroblastoma. Four years later, the patient remained disease‐free.

**FIGURE 3:**
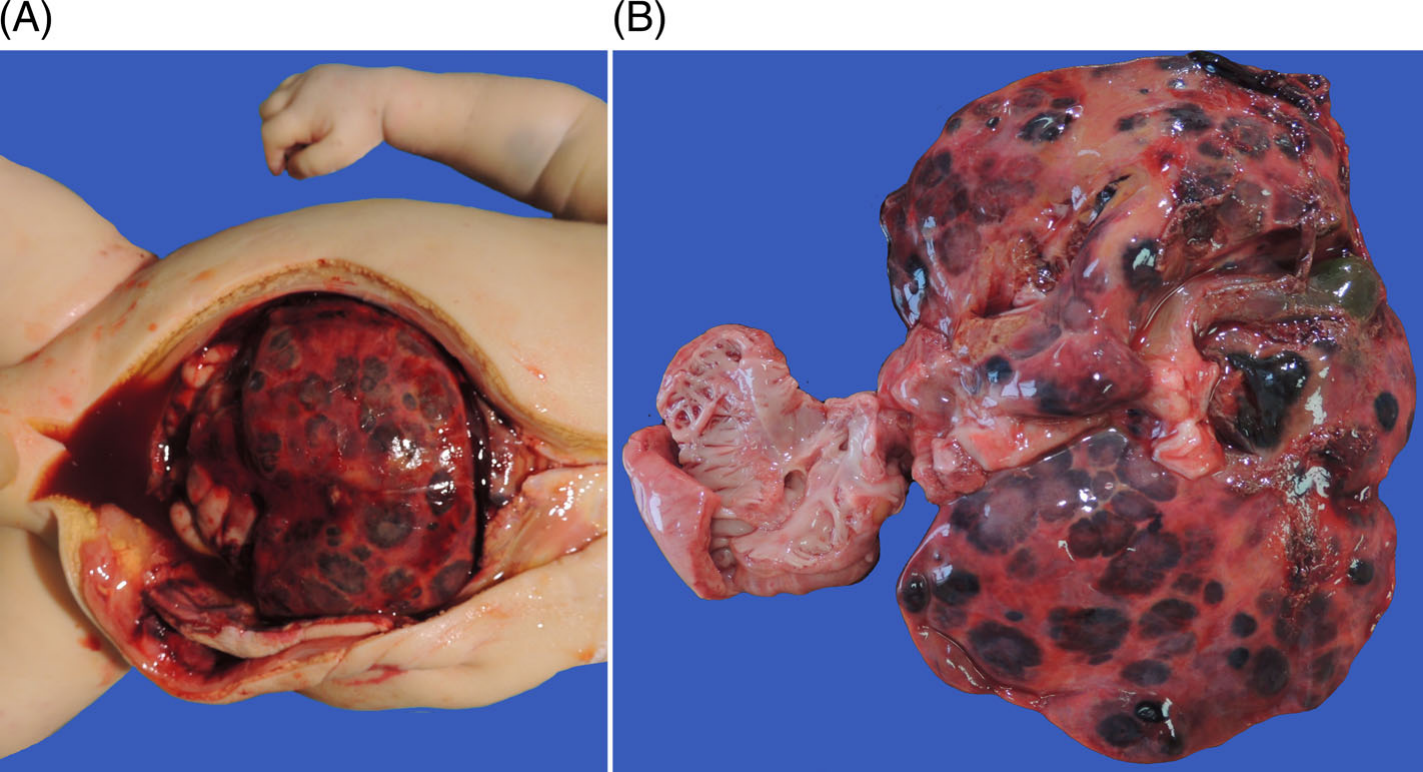
(A, B) Autoptic aspect of the liver in case 5. Front (A) and posterior (B) views indicate the extent of the disease. The arrows indicate foci of hemangioendothelioma. Flow Chart: diagram summarizing the principles of management. Note how the central node of the algorithm relies on whether or not diagnoses can be made

Case 5 A newborn boy delivered at 37 WoP and weighing 3160 g with a normal antenatal history was admitted to the neonatal intensive care unit at 10 days of life for vomiting, polypnea, and a rapidly increasing abdominal girth. MRI showed a large abdominal mass that probably originated from the liver and occupied the entire abdomen. In 2 h, the abdominal diameter increased from 38 to 40 cm. The alpha‐fetoprotein level was remarkably increased (13 354 mcg/L), the beta‐HCG level was 0.5 mcg/L, and levels of urinary catecholamines were within normal limits (VMA, 5.66 mmol/L; HVA, 5.62 mmol/L, HVA/creatinine, 3.16 mmol/L). The aspartate transaminase/alanine transaminase ratio was 2097:309. Kasabach–Merritt syndrome was suspected as hypofibrinogenemia (0.9 g/L), consumptive coagulopathy (Factor V, 20%; Factor VII, 14%), and disseminated intravascular coagulation were noted. Cytoreductive chemotherapy was initiated at the time of admission using corticosteroids (methylprednisolone 1 mg/kg) and completed with etoposide and carboplatin.^4^ Despite ventilator support, the patient's clinical status worsened, and decompressive laparostomy was performed at 18 days of life. Unfortunately, the newborn died 2 days after surgery. The pathological diagnosis was hepatic multifocal infantile hemangioma (Figure 3A,B).

## 4 DISCUSSION

### 4.1 Diagnosis and presentation

In newborns, large benign and malignant tumors found in the abdomen can originate from either peritoneal or retroperitoneal structures (mesenteric, bowel, liver, kidney, adrenal glands, and pancreas). These tumors are extremely rare; however, consequent acute decompensation with emergent symptomatology in newborns is even rarer.^1^

A rapidly growing abdominal mass may threaten the lives of newborns. For example, in 2015, Majjiga et al^5^ reported the case of a newborn without any antenatal abnormalities detected on routine US that underwent surgery at birth for a rapidly worsening general condition and significant abdominal distention. Emergency laparotomy was immediately performed, revealing an 110 × 90 × 50 mm cauliflower‐shaped tumor originating from the retroperitoneum. Histopathological examination revealed an immature teratoma with tumor elements in the microscopic yolk sac. Interestingly, the authors reported that “additional radiographic studies, such as computed tomography scanning or abdominal US, were not performed because of the infant's rapidly deteriorating condition. It was felt that her only chance of survival was immediate surgical intervention to obtain homeostasis.”

Prenatal diagnosis of solid abdominal masses has also been reported. For example, in a case series, De Backer et al^6^ reported a 150 × 150 × 150 mm immature teratoma diagnosed antenatally at 34 weeks of gestation. Upon delivery, the tumor was resected as it severely compromised the clinical status of the newborn. However, the newborn died a few hours after surgery.

Such examples, as in our own case series, emphasize the difficulties in making the most ideal decision. Furthermore, although there are protocols for each type of tumor,^4^, ^7^, ^8^, ^9^ it is neither possible nor safe to apply them without a confirmed diagnosis. The histopathological nature of the tumor can only be assumed in these situations, and this uncertainty leads to hesitation between immediate surgical removal, abdominal decompression, or medical treatment after stabilization. Although classical biological markers may be used, the results are not immediately known, especially when a newborn's clinical status rapidly worsens. Thus, two issues must be addressed: the optimal timing and type of intervention.

### 4.2 Indications for surgery

In our study, among the five newborns who underwent surgery, increasing need for ventilatory support was the primary indication for surgery in four newborns. Even though one of the masses (hepatic hemangioma in case three) could have been treated with systemic corticosteroids or propranolol,^10^ the newborn's rapidly worsening clinical condition meant that abdominal decompression was necessary.

Abdominal compartment syndrome is defined as “sustained elevation in intra‐abdominal pressure >10 mmHg associated with new or worsening organ dysfunction that can be attributed to elevated intra‐abdominal pressure.” Intra‐abdominal pressure is defined as “the steady‐state pressure concealed within the abdominal cavity at the end of expiration in the supine position, after ensuring that abdominal muscle contractions are absent and with the transducer zeroed at the level of the mild axillary line”.^11^, ^12^, ^13^ However, in our series, we did not routinely measure intra‐abdominal pressure nor did all the studies we reviewed.^14^, ^15^, ^16^

Another life‐threatening condition is Kasabach–Merritt phenomena.

In 2003, Bärtsch et al^17^ reported a case of giant hepatic hemangioma in a newborn with a prenatal diagnosis of an abdominal solid mass. In 2007, Francis^18^ also reported a case of a newborn diagnosed with a large hepatic mesenchymal hamartoma at birth.

Surgical removal of the tumor causing coagulopathy is associated with normalization of hematological abnormalities. However, due to evolving coagulopathy and size and infiltrative nature of the tumor, surgery is considered a high‐risk procedure that may cause bleeding.^19^

Hypertension is also an indication for early surgical treatment in newborns. Many reports have described abdominal neonatal masses that were surgically resected early due to non‐responsive hypertension. The masses were mostly renally or adrenally located such as in congenital mesoblastic nephroma or intrarenal neuroblastoma. As shown in the fourth case in our series, the baby underwent surgery for treatment‐resistant hypertension. His condition improved significantly shortly after surgery. In 2017, Robertson Bell reported a case of a baby diagnosed with congenital mesoblastic nephroma who, despite a high dose of hydralazine, had an average blood pressure that remained elevated. Surgical resection was performed at 2 days of life, and the renin level decreased 2 days postoperatively.^20^

Overall, the benefit of surgery in the absence of a definite diagnosis must be considered. Surgeons and pediatricians should share common variables and scoring systems to monitor the patients' clinical courses.

In 1996, Hsu et al published an article about a long case series of neuroblastomas,^21^ attempting to state that a retrospective semiquantitative scoring system could indicate when children require treatment. All seven neonates who died in the series had hepatomegaly at birth and died of either complications from liver enlargement or treatment. Interestingly, they also stated that an increase of >1 cm in the abdominal perimeter in less than 48 h calls for careful surveillance. In the absence of a valid scoring system, we suggest the following criteria be considered major in surgical decision‐making: (1) rapid increase in abdominal diameter; (2) emergent need for ventilatory support; and (3) low urine output or worsening NIRS, as previously reported^22^ (Table 2). As minor criteria, we suggest the presence of hypertension, impaired coagulation, collateral circulation, and the need for vasopressive support. In our opinion, the combination of two major criteria or one major and three minor criteria may serve as indications for surgery.

**TABLE 2:** Criteria in surgical decision making

MAJOR criteria	MINOR criteria
Abdominal diameter increased >1 cm in 24 h	Hypertension
Increase of ventilatory support in 8–12 h	Impaired clotting factors
Diuresis decreased below 1 cc/kg/h	Collateral circulation
NIRS parameters worsening in 6–8 h^20^	Need of vasopressive support

### 4.3 Surgical strategies

If a surgical option is chosen, the optimal form of intervention should be determined next. Based on our experience, choosing between laparostomy and laparotomy and excision was difficult. In the reviewed literature, laparoscopy seems to have no role in this rescue indication, and authors believe that there is insufficient space in the abdominal cavity, such as in cases of overdistention.

It is unclear whether decompression could lead to an improved prognosis because of the heterogeneity of the reports and the absence of comparative trials. In 2008, Harper et al^23^ reported two cases of hepatomegaly related to neuroblastoma that were successfully treated with a silo or patch using the open abdomen technique. Robert et al^24^ in 2002 reported a case of a two‐month‐old infant with 4S neuroblastoma complicated by massive hepatomegaly that was managed by abdominal decompression surgery and a negative pressure dressing system. Muller‐Berghaus et al used this technique in one case of disseminated neuroblastoma, and the patient survived.^15^ Unfortunately, the authors did not precisely describe the condition of the baby before surgery. Thus, determining when surgery can help decompress the abdominal compartment without adverse consequences for the child remains difficult. In the fourth case, we placed an Alexis® (Applied Medical Rancho Santa Margarita, California, CA, USA) retractor to provide room for decompression. It was effective in improving both circulation and ventilatory parameters but insufficient for the newborn's survival. The authors suggest that this technique should be used as a life‐saving procedure in a timely manner. In other words, laparostomies performed under critical conditions result in an extremely poor prognosis.

The discussion regarding laparostomy or excision versus biopsy and chemotherapy‐delayed surgery remains controversial. Furthermore, the individual presentation of patients requires tailored responses.

To summarize, we suggest following the management algorithm in flow chart n.1 based on our experiences in the management of these situations.

The strength of the study lies in trying to provide a clinical algorithm in front of a rare situation in which difficult decisions with potentially fatal outcomes for the patient need to be made quickly. In addition, an attempt was made to provide objective parameters to be calculated that can help in making a choice about whether or not to take surgical care.

This study has some limitations. Due to the rarity of the disease, heterogeneity of the reports in the literature, and a short case series, we could not draw conclusions based on the literature alone. Most importantly, we could not infer the statistics. Even with a standardized protocol for surgical management, multiple questions remain unanswered on whether the antenatal diagnosis can affect prognosis.

## 5 CONCLUSIONS

In conclusion, we should infer that:

1. In these cases, management should be based on the clinical status of the patient and not on the correct diagnosis; if the patient's condition worsens, decompression surgery should be considered even if the origin of the mass is unknown.
2. An increasing abdominal diameter, increasing need for ventilatory support, worsening urine output, and NIRS are the most important warning signs. If the liver is affected by the tumor and coagulopathy is present, prognosis is often compromised.
3. Selecting between the laparostomy and full excision requires a tailored approach and multidisciplinary evaluation.

## AUTHOR CONTRIBUTIONS

**Francesco Laconi:** Conceptualization (lead); data curation (equal); formal analysis (equal); investigation (equal); methodology (lead); project administration (equal); resources (equal); software (equal); supervision (equal); validation (equal); visualization (equal); writing – original draft (lead); writing – review and editing (lead). **Charline Bischoff:** Conceptualization (equal); data curation (lead); formal analysis (equal); investigation (equal); methodology (equal); project administration (equal); resources (equal); software (equal); supervision (equal); validation (lead); visualization (lead); writing – original draft (equal); writing – review and editing (equal). **Daphne Michelet:** Conceptualization (supporting); data curation (equal); formal analysis (equal); investigation (equal); methodology (supporting); project administration (equal); resources (equal); software (equal); supervision (supporting); validation (equal); visualization (equal); writing – original draft (supporting); writing – review and editing (supporting). **Gauthier Loron:** Conceptualization (supporting); data curation (equal); formal analysis (equal); investigation (equal); methodology (equal); project administration (equal); resources (lead); software (equal); supervision (supporting); validation (supporting); visualization (equal); writing – original draft (supporting); writing – review and editing (equal). **Nathalie Bednarek:** Conceptualization (supporting); data curation (equal); formal analysis (equal); investigation (equal); methodology (equal); project administration (supporting); resources (equal); software (equal); supervision (supporting); validation (equal); visualization (equal); writing – original draft (supporting); writing – review and editing (supporting). **Marie‐Laurence Poli‐Merol:** Conceptualization (equal); data curation (supporting); formal analysis (supporting); investigation (supporting); methodology (supporting); project administration (lead); resources (supporting); software (supporting); supervision (lead); validation (lead); visualization (supporting); writing – original draft (equal); writing – review and editing (lead). **Maguelonne Pons:** Conceptualization (equal); data curation (equal); formal analysis (equal); investigation (equal); methodology (equal); project administration (equal); resources (equal); software (lead); supervision (equal); validation (equal); visualization (equal); writing – original draft (supporting); writing – review and editing (equal). **Nadia Boudaoud:** Conceptualization (equal); data curation (equal); formal analysis (equal); investigation (equal); methodology (equal); project administration (lead); resources (lead); software (lead); supervision (lead); validation (lead); visualization (lead); writing – original draft (equal); writing – review and editing (equal).

## CONFLICT OF INTEREST

The authors declare that they have no conflict of interest.

## ETHICS STATEMENT

All procedures performed in this study involving human participants were in accordance with the ethical standards of the institutional and/or national research committee and with the 1964 Helsinki declaration and its later amendments or comparable ethical standards. Informed consent was obtained from all participants included in the study.

## Data Availability

Data sharing is not applicable to this article as no new data were created or analyzed in this study.
